# From Waste to Value: Producing a Non-Astringent Persimmon Paste from Discarded ‘Rojo Brillante’ Fruit

**DOI:** 10.3390/foods15193522

**Published:** 2026-10-01

**Authors:** Virginia Larrea, Alexandre Tormos, Isabel Hernando, Gemma Moraga

**Affiliations:** Instituto Universitario de Ingeniería de Alimentos–FoodUPV, Universitat Politècnica de València, Camino de Vera, s/n, 46022 Valencia, Spain; virlarsa@tal.upv.es (V.L.); altoril@alumni.upv.es (A.T.); gemmoba1@tal.upv.es (G.M.)

**Keywords:** astringency, *Diospyros kaki* Thunb., food waste reduction, sensory analysis, soluble tannins, thermal processing

## Abstract

‘Rojo Brillante’ persimmon has positioned Spain as the second largest producer worldwide. Its rapid expansion has increased seasonal overproduction and postharvest losses, particularly of astringent fruit unsuitable for fresh markets; however, the high soluble tannin content of this cultivar limits processing opportunities. In this study, a persimmon paste was developed from astringent discarded fruit. The combined effects of thermal treatment (70 and 100 °C) and sugar concentration (30 and 50%, *w/w*) on soluble tannin content and physicochemical properties were evaluated, together with their evolution during refrigerated storage. A ranking test was conducted to assess the astringency and other sensory attributes among formulations. Processed pastes contained a maximum of 1.01 mg GAE/g, compared with 7.3 mg GAE/g in fresh fruit, showing a decrease in the values measured by the Folin–Ciocalteu assay during processing. Importantly, these values did not increase significantly during refrigerated storage. Formulations with 50% sugar exhibited the lowest soluble tannin contents, with no significant differences attributable to thermal treatment. In contrast, samples with 30% sugar developed firmer gel structures, showing higher mechanical resistance. Pastes processed at 100 °C presented improved color stability during storage, likely associated with inactivation of enzymatic browning. Positive purchase intention was 70%, while the 100 °C/50% formulation was the most frequently selected for purchase (60%). These findings demonstrate the potential of persimmon paste as a sustainable valorization pathway for astringent ‘Rojo Brillante’ discarded fruit.

## 1. Introduction

Persimmon (*Diospyros kaki* Thunb.) has become one of the most economically relevant fruit crops in Mediterranean production systems. In Spain, the cultivar ‘Rojo Brillante’ has driven a remarkable expansion of the sector over the last two decades, positioning the country as the second largest persimmon producer worldwide after China [1]. Production is highly concentrated in the Valencian Community under the Protected Designation of Origin (PDO) “Kaki Ribera del Xúquer”, and the introduction of industrial deastringency treatments has been the key technological factor enabling commercialization of firm fruit [2,3]. Despite its commercial success, rapid expansion has led to increased seasonal overproduction and postharvest losses, particularly of fruit unsuitable for fresh markets, due to mechanical damage, physiological disorders, or incomplete deastringency. The development of stable value-added products from surplus fruit represents a strategic pathway to enhance sustainability and reduce waste within the persimmon value chain. However, technological diversification of ‘Rojo Brillante’ remains constrained by its intrinsic astringency. Persimmon cultivars are classified according to their astringency behavior into pollination-constant non-astringent (PCNA), pollination-variant non-astringent (PVNA), pollination-constant astringent (PCA), and pollination-variant astringent (PVA) types. ‘Rojo Brillante’ belongs to the PVA group and accumulates high concentrations of soluble tannins (proanthocyanidins) during fruit development [4]. Although soluble tannin levels progressively decrease during ripening, ‘Rojo Brillante’ is harvested at a firm stage to facilitate handling, transport, and distribution while minimizing mechanical damage and postharvest losses. At this stage, however, the fruit remains astringent. Astringency perception is associated with the interaction between soluble tannins and salivary proteins, particularly proline-rich proteins, leading to protein aggregation and reduced oral lubrication, perceived as dryness and roughness [5]. Therefore, the soluble fraction of tannins, rather than total phenolic content, determines the sensory impact.

Commercial removal of astringency is currently achieved through several postharvest techniques, among which exposure to high CO_2_ atmospheres (typically 95–98%) under controlled temperature and relative humidity conditions is the most widely applied at industrial scale [2]. These anaerobic conditions induce acetaldehyde accumulation via fermentative metabolism, promoting tannin polymerization and insolubilization without excessive fruit softening. Previous studies have demonstrated that acetaldehyde-mediated crosslinking leads to the formation of insoluble tannin complexes within tannin cells, effectively reducing soluble tannin content [2,6]. However, the effectiveness of deastringency treatment may be compromised by fruit maturity, as physiological and microstructural changes during ripening can limit the fruit’s capacity for tannin insolubilization [6].

While deastringency enables fresh consumption, processing introduces additional technological challenges. Thermal treatments applied during pasteurization or concentration may promote partial re-solubilization of previously insolubilized tannins, leading to recurrence of astringency in derived products [7,8]. Castelló et al. [7] demonstrated that heat treatment during filling and storage of persimmon spreads influenced soluble tannin levels and sensory perception. Moreover, temperature conditions can modulate tannin stability, and astringency recurrence has been associated with both processing intensity and storage temperature [9]. Matrix composition also plays a decisive role in modulating astringency. Beyond acetaldehyde-mediated polymerization, soluble tannins can interact with structural polysaccharides, particularly pectins, promoting their insolubilization [10]. In addition, pectins can interfere with tannin–salivary protein interactions through hydrogen bonding and hydrophobic interactions, favoring the formation of more soluble protein–tannin–pectin complexes and thereby reducing salivary protein precipitation and astringency perception [5]. Furthermore, sweet-tasting sugars can mitigate astringency perception in food systems [11]. Therefore, formulation variables, including sugar and pectin content, may significantly influence tannin solubility and sensory stability in processed persimmon products.

From a technological perspective, the development of a persimmon paste analogous to quince paste represents a promising valorization strategy. Traditional quince paste is characterized by a firm gel structure with high soluble solids, reduced water activity, and acidic pH that ensures extended shelf life [12]. Replicating these characteristics in persimmon matrices requires careful control of sugar concentration, acidification, and thermal treatment. Sugar concentration not only determines sweetness perception but also affects water activity, gel structure, and molecular crowding within the matrix. High soluble solids environments may limit tannin mobility and re-solubilization, whereas lower sugar formulations contain a higher fruit proportion and therefore potentially higher initial soluble tannin levels.

Refrigerated storage is frequently applied to preserve microbiological stability and sensory attributes in fruit-based products. Nevertheless, storage is not a passive stage. Studies on persimmon spreads and paste matrices have reported color shifts, structural modifications, and changes in phenolic fractions during storage [7,13]. In some cases, partial re-solubilization of tannins has been observed even in previously deastringed material [8]. These findings underline the importance of evaluating both immediate processing effects and temporal evolution of tannins during storage. Moreover, the combined influence of processing variables such as thermal intensity and sugar concentration on soluble tannin content and product quality has received limited attention. In this context, information on quince-type persimmon pastes, particularly regarding their stability during refrigerated storage, remains limited.

Therefore, the objective of the present study was to evaluate the influence of thermal treatment and sugar concentration on soluble tannin content, physicochemical properties (pH, soluble solids, water activity, color, and texture), and sensory attributes of persimmon paste produced from astringent ‘Rojo Brillante’ fruit. In addition, the evolution of these parameters during refrigerated storage was assessed to determine the stability of tannin insolubilization and overall product quality. By considering formulation and processing variables alongside storage behavior, this work aims to contribute to the development of non-astringent, value-added persimmon products capable of reducing postharvest losses and improving sustainability in the persimmon sector.

## 2. Materials and Methods

### 2.1. Sample Preparation

Astringent persimmons of the ‘Rojo Brillante’ variety were used. These persimmons, coming from culls, were supplied by the Cooperativa Agrícola de Catadau S. COOP. LTDA (Catadau, Spain). In total, four different persimmon paste formulations were produced by combining two processing temperatures, 70 or 100 °C, with two sugar levels, 30 or 50% (*w/w*). These percentages refer to the proportion of sugar in the initial persimmon–sugar mixture. Lemon juice was added to achieve a pH below 4, which guarantees microbiological stability against several of the main pathogens. Preparation began by blending the ingredients in a Thermomix^®^ TM6 (Vorwerk, Wuppertal, Germany) for 30 s using a progressive speed from 5 to 10. The mixture was then cooked at either 70 or 100 °C, at speed 5, until reaching 65 °Brix. This processing endpoint was selected to obtain products with a comparable final soluble solids concentration. Therefore, differences between treatments should be interpreted in terms of the temperature–time conditions required to reach this endpoint.

Four persimmon paste treatments were obtained: 70 °C with 30% sugar (70/30), 70 °C with 50% sugar (70/50), 100 °C with 30% sugar (100/30), and 100 °C with 50% sugar (100/50). Two independent production replicates were prepared for each formulation. Each production replicate was divided into separate containers for each storage time and stored at 4 °C for 2 months. At each storage time (0, 15, 30, and 60 days), one container from each production replicate was analyzed. Physicochemical measurements were performed in triplicate for each container.

### 2.2. pH

pH was measured using a calibrated digital pH meter (pH-meter 20+, Crison, Alella, Spain) equipped with a combined glass electrode and temperature probe. The electrode was directly inserted into the sample.

### 2.3. Total Soluble Solids

The total soluble solids were measured using a thermostated digital refractometer (RFM330+, Bellingham and Stanley Ltd., Tunbridge Wells, UK) at 20 °C. Samples were homogenized prior to refractometric measurement. Results were expressed as °Brix.

### 2.4. Water Activity

The water activity was measured using a water activity meter (WaterLab, Steroglass, Perugia, Italy).

### 2.5. Soluble Tannin Content

Soluble tannins were extracted from 5 g of sample using 96% ethanol. The mixture was homogenized using an Ultra-Turrax (IKA, Staufen, Germany) for 1 min at 12,000 rpm. After centrifugation (10,000 rpm, 20 min, 4 °C), the supernatant was filtered and collected. The extraction procedure was repeated twice, and combined extracts were adjusted to 100 mL with 96% ethanol in a volumetric flask. Extracts were stored in opaque containers at −18 °C until tannin determination. Two independent extracts were prepared from the fresh persimmon fruit (raw material) and from each persimmon paste formulation. All extracts were analyzed after 1 week of storage (−18 °C).

The soluble tannin content was determined using the Folin–Ciocalteu method. Prior to analysis, the extracts were thawed under chilled conditions. Each extract was mixed with distilled water (7:1), and 0.5 mL of Folin–Ciocalteu reagent was added at room temperature. After 3 min, 1 mL of 20% Na_2_CO_3_ and 1.5 mL of distilled water were added. The mixture was kept in the dark for 90 min. After this period, the absorbance was measured at 725 nm using a Helios Zeta UV-Vis spectrophotometer (Thermo Fisher Scientific Inc., Waltham, MA, USA). A gallic acid calibration curve (0–150 ppm) was used, and the results were expressed as mg gallic acid equivalents (GAE) per g of sample.

### 2.6. Color

A Chroma Meter CR-400 (Konica Minolta, Tokyo, Japan) with a D65 illuminant and 10° standard observer was used to obtain the CIE L*a*b* color coordinates, where L* represents lightness on a scale from 0 (black) to 100 (white), a* (+) red or (−) green, and b* (+) yellow or (−) blue [8]. An optical glass was used to avoid direct contact with the sample. From the obtained coordinates, hue angle (h*) and chroma (C*) were calculated using Equations (1) and (2), respectively. The total color difference (ΔE*) was calculated by Equation (3) using the initial state of each formulation as reference.

(1) $$h^{*}=\tan^{-1}\frac{b^{*}}{a^{*}}$$

(2) $$C^{*}=\sqrt{a^{*2}+b^{*2}}$$

(3) $${∆E}^{*}=\sqrt{{\left(L^{*}-L_{0}^{*}\right)}^{2}+{\left(a^{*}-a_{0}^{*}\right)}^{2}{\left(b^{*}-b_{0}^{*}\right)}^{2}}$$

### 2.7. Texture

Texture was evaluated using a TA-XT Plus Texture Analyzer (Stable Micro Systems, Ltd., Godalming, UK) by performing a puncture test. A cylindrical Delrin probe (P/0.5R) was used at a test speed of 1 mm/s until reaching a penetration distance of 5 mm after registering a trigger force of 0.049 N. During the return phase, the probe moved back to its initial position at the same speed. Rupture force (g), maximum force (g), and adhesiveness (g·s) were analyzed.

### 2.8. Sensory Analysis

A ranking test was conducted according to Castelló et al. [7].Twenty semi-trained panelists evaluated the four formulations after 15 days of storage. The assessed attributes were visual appearance, caramel color, astringency, flavor preference, sweetness, texture preference, and global evaluation. For each attribute, panelists ranked the samples from 1 to 4, where 1 corresponded to the lowest intensity and 4 to the highest intensity, and the results were expressed as the mean rank assigned by the 20 panelists to each formulation. Additionally, the 20 panelists were asked to indicate their purchase intention (yes/no/undecided) and to select the sample they would most likely purchase. Results were expressed as percentages of responses.

### 2.9. Statistical Analysis

Data were statistically analyzed using Statgraphics Centurion XIX software, version 19.1.2 (StatPoint Technologies, Inc., Warrenton, VA, USA). For the physicochemical parameters, separate one-way analyses of variance (ANOVA) were performed. To evaluate the effect of storage time, each formulation was analyzed independently using storage time as the factor. To evaluate differences among formulations, a separate one-way ANOVA was performed at each storage time using formulation as the factor. The triplicate physicochemical measurements from the two independent production replicates were not averaged prior to statistical analysis; all six measurements were included in the ANOVA (n = 6). Fisher’s least significant difference (LSD) test was used for multiple comparisons, with significance set at *p* < 0.05. Sensory ranking data were analyzed using the Friedman test, considering panelists as blocks and formulation as the treatment factor, with significance set at *p* < 0.05.

## 3. Results

### 3.1. pH

pH is an important parameter to control, as an acidic pH prevents the proliferation of spoilage microorganisms. In fruit-based products, pH and acidity are also recognized as fundamental variables for defining overall physicochemical stability and characterizing product quality during storage [14]. Table 1 shows the pH values for each formulation, as well as their evolution during refrigerated storage.

The initial pH of all persimmon paste formulations was below 4, as lemon juice acidification reduced the natural pH of persimmon from its typical range of 5.5–6.0, depending on maturity stage and seasonal conditions [7,15]. This acidification is consistent with the requirements for fruit-based gelled products, which are commonly formulated within an acidic range to ensure both microbiological stability and proper gel formation [16]. Among the treatments, the 100/50 formulation exhibited the lowest initial pH, whereas the 70/30 formulation showed the highest value, with significant differences between them (*p* < 0.05). These differences can be attributed to the combined effect of processing temperature and sugar concentration. Heating at 100 °C may enhance the release and solubilization of organic acids or promote hydrolytic reactions that increase the availability of acidic compounds in the matrix, resulting in a lower pH. Additionally, the higher sugar concentration (50%) may alter solute–solvent interactions and affect acid dissociation equilibria, thereby contributing to the observed decrease in pH [17].

All samples exhibited a similar trend during storage, with a significant increase (*p* < 0.05) in pH up to day 30, followed by a significant decrease (*p* < 0.05) at the end of storage. This fluctuation contrasts with findings in blueberry jam research, where no significant interaction was found between storage time and pH values, showing a more stable profile over time [14]. In our samples, the initial increase may be related to chemical transformations affecting organic acids within the high-sugar matrix, such as partial degradation, interactions with polysaccharides or phenolic compounds, or shifts in acid dissociation equilibria, which could temporarily reduce the concentration of free protons.

Nevertheless, from a microbiological safety standpoint, all formulations remained within the acidic range throughout storage. Although a slight increase in pH was observed during storage, all the samples maintained pH values below 4.6, the conventional threshold used to classify acid foods and to prevent the growth of *Clostridium botulinum* under normal conditions [18,19]. In addition, the pH values remained well below the optimum range reported for the growth of *Escherichia coli*, which generally occurs near neutral pH values [20].

### 3.2. Total Soluble Solids and Water Activity

Figure 1 shows the evolution of total soluble solids (°Brix) and water activity (a_w_) in the four persimmon paste formulations during refrigerated storage.

As expected, all formulations reached total soluble solids values close to the target level of 65 °Brix after processing. During refrigerated storage, a decreasing trend in °Brix values was observed in all formulations. Changes in soluble solids during storage have been previously reported in fruit-based products and are generally attributed to physicochemical modifications within the matrix, including water redistribution, structural rearrangements, and interactions among soluble components such as sugars, organic acids, and polysaccharides [14,21]. In addition, partial sugar crystallization during storage could have reduced the fraction of sugars remaining dissolved in the liquid phase, thereby contributing to the lower °Brix values. Therefore, the decrease observed in this study is more likely related to changes in the distribution and availability of soluble solids rather than to the degradation of specific compounds. At the end of storage, formulations with 50% sugar maintained significantly higher (*p* < 0.05) °Brix values than those with 30% sugar.

Regarding a_w_, initial values ranged between 0.815 and 0.855, showing significant differences depending on formulation. The 70/30 formulation presented the highest a_w_ value (*p* < 0.05), while 70/50 showed the lowest (*p* < 0.05). No significant differences (*p* > 0.05) were observed between 100/30 and 100/50. In general, during refrigerated storage, the decreasing trend in °Brix led to an increasing trend in a_w_ values. This is consistent with the inverse relationship between soluble solids concentration and water activity, as higher sugar content reduces the availability of free water through osmotic effects and water-binding phenomena [14,22]. After 60 days of storage, formulation 100/30, which had the lowest soluble solids content, presented the highest water activity value (a_w_~0.871), supporting the strong dependence of a_w_ on the concentration of soluble solids.

### 3.3. Soluble Tannin Content

The soluble tannin content, responsible for the astringency of the product, ranged from 0.44 to 1.01 mg GAE/g (Figure 2). These values were markedly lower than the 7.3 mg GAE/g measured in the fresh persimmon fruit used to formulate the products. The initial tannin concentration confirmed the astringent nature of the fruit prior to processing and was consistent with values previously reported for astringent ‘Rojo Brillante’ persimmon at an intermediate maturity stage [2].

The observed reduction in soluble tannins during processing can be explained by several mechanisms promoting tannin insolubilization. During processing, tissue disruption releases soluble tannins from tannin cells, increasing their contact with cell wall material. They may subsequently adsorb onto cell wall fragments and associate with pectic polysaccharides through non-covalent interactions, including hydrogen bonding and hydrophobic forces, promoting the formation of less soluble tannin-associated structures [10,23]. Thermal processing may further influence these tannin–matrix interactions. Recent evidence from proanthocyanidin–pectin systems demonstrated that heating can promote covalent binding between proanthocyanidins and pectin through the formation of proanthocyanidin–pectin adducts, providing a potential additional mechanism for the stabilization of tannins within the polysaccharide matrix [24]. However, thermal treatment of astringent persimmon does not necessarily result in a non-astringent product. The final response depends on the initial soluble tannin content, the composition and structure of the matrix, the availability of tannin-binding polysaccharides, and the extent of concentration or water removal during processing. Taboada et al. [8] showed that astringent and non-astringent ‘Rojo Brillante’ behaved differently after processing into cremogenates, while the effect of adding thickeners depended on the initial astringency of the fruit. They reported that cremogenates prepared directly from astringent fruit remained highly astringent after pasteurization, even when pectin, sodium alginate, or guar gum was added. Tsurunaga et al. [13] also demonstrated that the effect of heating on persimmon paste depends strongly on the heating method: boiling and pressurized heating favored astringency recurrence in previously deastringed paste, whereas stir-frying, which was accompanied by moisture evaporation, was more effective in maintaining low astringency.

The importance of tannin–polysaccharide interactions in processed persimmon matrices is further supported by Tsurunaga et al. [25]. In astringent persimmon paste, these authors showed that the addition of several polysaccharides, including high- and low-methoxyl pectins, carrageenan, xanthan gum, and sodium alginate, markedly reduced astringency and soluble tannin content. Moreover, high- and low-methoxyl pectins and sodium alginate prevented astringency recurrence after thermal treatment, supporting the role of tannin–polysaccharide interactions in stabilizing tannins in insoluble forms. These mechanisms are expected to be particularly relevant in concentrated fruit matrices, where tissue disruption and water removal during processing increase the exposure of tannins to structural polysaccharides, favoring the formation of insoluble complexes.

Two groups were clearly observed from day 0, with higher soluble tannin levels (0.96–1.01 mg GAE/g) in formulations with higher fruit content (70/30 and 100/30) and lower values (0.44–0.45 mg GAE/g) in formulations with higher sugar content (70/50 and 100/50). Since soluble tannins were expressed per gram of product, the lower values observed in the high-sugar formulations are associated with the lower proportion of persimmon in the final matrix. To further assess this dilution effect, soluble tannin values at day 0 were normalized to the initial amount of persimmon used in each formulation. A mass balance was applied based on the initial soluble solids content of the fruit, the formulation ratio, and the final soluble solids concentration. The mean normalized soluble tannin content was 0.83 ± 0.03 mg GAE/g initial persimmon, with no significant differences (*p* > 0.05) among formulations. This result supports that the apparent differences observed on a product-weight basis were mainly associated with formulation dilution rather than with differences in tannin extractability. This is consistent with previous reports showing that sucrose does not play a direct role in tannin insolubilization; however, higher sugar concentrations may contribute to a lower perception of astringency through sweetness-mediated masking effects and changes in matrix viscosity, both of which can influence oral lubrication and reduce the sensory impact of tannins [23].

Interestingly, the soluble tannin levels obtained in the present study were lower than those previously observed in a similar persimmon paste developed within our research group from CO_2_-treated non-astringent persimmon processed at 100 °C with 50% sugar [26]. In that study, the final product exhibited a marked astringency and a soluble tannin content of 1.15 mg GAE/g. These results suggest that the initial physicochemical state of tannins may strongly influence their behavior during thermal processing. In CO_2_-treated fruit, tannins are previously insolubilized through acetaldehyde-mediated aggregation and retained in a non-astringent persimmon [2]. However, an intense thermal processing may alter the stability of these aggregates, promoting partial re-solubilization and astringency recurrence, as reported in other processed persimmon products [7,9,13]. In contrast, when astringent fruit was used as raw material in the present study, the product processed at 100 °C and the same sugar concentration (50%), showed the lowest soluble tannin content (0.44 mg GAE/g). Therefore, the markedly different final soluble tannin contents of the two products are consistent with two different processing pathways: thermal destabilization and partial re-solubilization of pre-existing insoluble tannin structures in previously deastringed fruit, versus progressive association of initially soluble tannins with the disrupted and increasingly concentrated polysaccharide-rich matrix when astringent fruit is processed directly.

During storage, all formulations exhibited a similar pattern, with an initial decrease in soluble tannins at day 15, followed by partial recovery and subsequent stabilization up to day 60. The early decrease may reflect continued post-processing equilibration and structural rearrangement of the concentrated matrix, during which tannin associations with pectins and other cell wall components may continue to evolve. Although significant increases (*p* < 0.05) in soluble tannin content were observed after day 15, no significant differences (*p* > 0.05) were found between the initial (day 0) and final (day 60) values. Therefore, no evidence of progressive tannin re-solubilization was detected during storage. The overall stability of the soluble tannin fraction suggests that the physicochemical conditions established during processing may have limited tannin re-solubilization during storage, potentially favored by the highly concentrated and polysaccharide-rich nature of the matrix [26].

From a technological perspective, these findings are particularly relevant because they demonstrate that quince-style persimmon paste can overcome one of the principal limitations traditionally associated with the processing of astringent persimmon fruit. While astringency recurrence has been repeatedly reported in thermally processed persimmon beverages, juices and pastes [7,9,13,27], the products developed in the present study maintained a low soluble tannin content. Therefore, this type of persimmon product represents a promising strategy for the valorization of astringent persimmon cultivars.

### 3.4. Color

Color stability is another key quality attribute influencing consumer acceptance of fruit-based products. Both enzymatic and non-enzymatic browning reactions may contribute to color changes during processing and storage. Enzymatic browning is mainly mediated by polyphenol oxidase (PPO) and peroxidase (POD), whereas non-enzymatic browning reactions, including Maillard reactions and caramelization, may develop during prolonged heating, particularly in high-sugar systems [28]. The extent of these reactions depends on processing conditions such as temperature and time, as well as on matrix composition. Figure 3 shows the position of the samples in the chromatic diagram and their evolution during refrigerated storage.

In this figure, hue angle (h*) is determined by the angle formed with respect to the b* = 0 axis and positive a* axis, where h* = 90° corresponds to a pure yellow tone and h* = 0° to a pure red tone. Chroma (C*) corresponds to the distance from the center (a* = 0, b* = 0) of the a* and b* coordinates, with pure colors located at the outer edge of the chromatic diagram. Moreover, in the CIE L*a*b* color space, there is a third axis where Lightness (L*) is represented, ranging from 0 (black) to 100 (white). Table 2 shows the values of the color attributes obtained for the four persimmon pastes and their evolution during refrigerated storage. Prior to storage (day 0), the lightness values (L*) of formulations 70/50 and 100/30 were significantly higher (*p* < 0.05) than those of the other samples, whereas 70/30 showed a significantly lower value (*p* < 0.05). Persimmon pastes 70/50 and 100/50 exhibited hue angle values of approximately 83°, close to 90°, indicating a predominantly yellow tone. In contrast, formulations 70/30 and 100/30, with hue angles of 62° and 68°, respectively, tended toward orange-reddish tones. This difference could be attributed to the higher proportion of persimmon in the 30% sugar formulations, which provided a greater amount of carotenoid pigments responsible for the orange-reddish color of the fruit [29]. Regarding chroma, all four formulations showed low color saturation or purity, particularly those with higher sugar content.

Throughout storage, all formulations exhibited perceptible color changes (ΔE* > 1.5) compared to their initial state. The greatest total color difference value (ΔE* = 9.5) was observed in formulation 70/30 after 15 days of storage, associated with a significant increase (*p* < 0.05) in L* and a significant decrease (*p* < 0.05) in C* and h*, resulting in a lighter, less saturated color, with a shift towards a more reddish-orange hue. Formulation 70/50 also showed a marked color difference after 60 days of storage (ΔE* = 7.0), with a significant decrease (*p* < 0.05) in L* and h*, indicating a darker color shifted toward a more orange hue. In general, samples subjected to 100 °C, particularly those with higher sugar content, showed smaller overall color differences. This behavior may be attributed to a greater degree of enzyme inactivation at higher temperatures, reducing the contribution of enzymatic browning reactions during storage [30].

### 3.5. Texture

Texture development in fruit-based pastes is largely determined by the structural characteristics and functionality of native cell wall polysaccharides, particularly pectins, whose physicochemical properties may be modified during processing. Pectin structure–function relationships are known to influence the mechanical properties of plant-based matrices, while thermal and non-thermal processing can alter pectin structure, functionality and, consequently, product properties [31,32,33]. In persimmon, pectins extracted from the ‘Rojo Brillante’ cultivar have been characterized by high molecular weight and high galacturonic acid content, indicating their potential contribution to matrix formation in concentrated products [33]. In addition, studies on persimmon-based semi-solid products have shown that formulation, thickener composition, thermal treatment and storage conditions significantly affect mechanical properties, syneresis and physical stability [7,8,13,25].

Figure 4 shows the evolution of rupture force, maximum force and adhesiveness of the four persimmon paste formulations during refrigerated storage. All formulations, except 70/50, exhibited a rupture peak characteristic of a structured gel-like behavior. As shown in Figure 4, formulations with 30% sugar exhibited significantly higher (*p* < 0.05) rupture force, maximum force and adhesiveness (greater negative values) than those formulated with 50% sugar. These results indicate that increasing the proportion of fruit promoted the formation of a stronger and more cohesive matrix. The higher fruit content likely increased the contribution of native cell wall material to the structural organization of the product, resulting in greater resistance to deformation and stronger adhesion to the probe during withdrawal. Under the conditions evaluated, this effect appeared to outweigh the increase in soluble solids, resulting in mechanically weaker structures despite the higher sugar content.

The influence of thermal treatment became evident when formulations with identical fruit-to-sugar ratios were compared. Formulation 70/30 exhibited significantly higher (*p* < 0.05) rupture force, maximum force and adhesiveness than formulation 100/30, demonstrating that processing temperature had a marked effect on the mechanical properties of the final product. Thermal processing is known to modify the physicochemical properties of pectic polysaccharides through solubilization and depolymerization reactions, thereby influencing the texture of fruit-based matrices [32]. Therefore, the differences observed between the two formulations may reflect differences in the extent of structural modifications occurring during processing. Interestingly, this trend was not maintained in the 50% sugar formulations. Formulation 70/50 was the only sample that did not exhibit a rupture peak, whereas formulation 100/50 developed a weak but measurable gel-like structure. This suggests that the higher processing temperature may have promoted partial matrix concentration or organization, but it was insufficient to compensate for the lower proportion of fruit-derived structural components in the 50% sugar formulations. Overall, these results indicate that fruit concentration exerted a greater influence on texture development than sugar concentration or thermal treatment considered independently.

Although significant differences were observed at the intermediate storage time (30 days), particularly in the formulations with 30% sugar, these changes were not sustained throughout storage. By the end of the refrigerated period (60 days), no significant differences (*p* > 0.05) were detected relative to the initial values, indicating that the mechanical properties remained essentially stable. These transient changes may reflect post-processing rearrangements within the concentrated matrix, involving water redistribution and changes in intermolecular interactions among cell wall polysaccharides [32,33]. The more pronounced changes observed in the 30% sugar formulations are consistent with their initially stronger matrix, which may have undergone a greater degree of structural relaxation before reaching equilibrium.

From a technological perspective, these findings indicate that quince-style persimmon paste can develop a stable and cohesive structure without the addition of external gelling agents.

### 3.6. Sensory Analysis

Sensory evaluation complements instrumental characterization by assessing whether the physicochemical differences among formulations are perceptible to panelists. The ranking test revealed significant differences (*p* < 0.05) among formulations for all evaluated attributes, including visual appearance, caramel color, astringency, flavor preference, sweetness, texture preference, and global evaluation (Figure 5).

Formulations 100/50 and 70/50 received the highest rankings for visual appearance, flavor, sweetness, and global evaluation. These results indicate that increasing sugar concentration enhanced sweetness perception and contributed positively to the sensory balance of the product. Sweetness is recognized as an important driver of food acceptance because it enhances flavor perception and mitigates the perception of astringency [11,25]. Formulation 70/30 received the highest ranking for texture preference, indicating that its textural characteristics were the most appreciated by the panelists under the conditions evaluated. However, this formulation and 100/30 were ranked as more astringent, in agreement with their significantly higher soluble tannin contents (Figure 2). This supports the well-established role of soluble tannins as the main contributors to astringency perception through their interaction with salivary proteins, leading to the characteristic sensations of dryness and roughness in the oral cavity [11]. Similar relationships between soluble tannin content and sensory astringency have also been reported in processed persimmon products, where thermal processing and matrix composition strongly influence the persistence of astringency during storage [7,25]. These formulations also received higher rankings for caramel color, consistent with the lower hue angle values observed instrumentally (Table 2), but lower rankings for visual appearance, flavor, sweetness and global evaluation. Regarding texture preference, formulation 70/30, which exhibited the highest rupture force and maximum force values (Figure 4), also received the highest sensory ranking for texture preference. In contrast, formulation 100/30, despite its mechanical properties, was the least ranked sample. This result suggests that sensory texture preference was not determined exclusively by mechanical resistance, but also by other oral attributes such as spreadability and mouthfeel. This discrepancy between instrumental and sensory measurements may be explained by the perception of a more granular structure in 100/30 formulation, which negatively affected mouthfeel. Similar divergences between instrumental parameters and sensory perception have been reported in fruit-based systems, where structural complexity plays a key role in texture acceptance [8].

Overall, the persimmon paste showed a high level of acceptance among the panelists, with 70% expressing a positive purchase intention (Figure 6A). Although purchase intention was assessed using a semi-trained panel rather than a consumer panel, these results indicate a favorable global perception of the product. The purchase intention observed in the present study was higher than that reported by Castelló et al. [7] for thermally processed persimmon spreads. In their study, pasteurization (90 °C, 20 min) markedly reduced product acceptability compared with the untreated product, which the authors associated with astringency recurrence and quality deterioration during processing. Regarding formulation preference, panelists most frequently selected formulation 100/50, followed by 70/50, whereas formulation 100/30 was not selected (Figure 6B). This preference was consistent with the ranking test, confirming that the formulations with 50% sugar achieved the highest global sensory acceptance. Similar trends have been reported for persimmon-derived products, where sweetness and reduced astringency are recognized as the main drivers of acceptance [34].

## 4. Conclusions

This study shows that stable persimmon pastes with high consumer acceptance can be successfully produced from astringent ‘Rojo Brillante’ persimmons. Thermal processing resulted in a substantial reduction in soluble tannin content, despite the high initial concentration characteristic of this cultivar, yielding products with soluble tannin concentrations low enough to markedly reduce the perception of astringency by consumers. Importantly, soluble tannin concentrations remained stable throughout refrigerated storage. Sugar concentration and processing temperature influenced the physicochemical characteristics of the pastes. Higher sugar concentration promoted a brighter yellow–orange color and improved sensory acceptance, whereas processing at 100 °C enhanced color stability, likely through greater inactivation of enzymes involved in enzymatic browning. Among the formulations evaluated, the paste processed at 100 °C with 50% sugar (100/50) provided the best balance between physicochemical stability and consumer acceptance, achieving the highest sensory scores and purchase intention. Overall, the proposed process represents a promising strategy for the valorization of astringent persimmons unsuitable for the fresh market, providing a stable, consumer-accepted product while contributing to the reduction in postharvest losses and food waste.

## Figures and Tables

**Figure 1:**
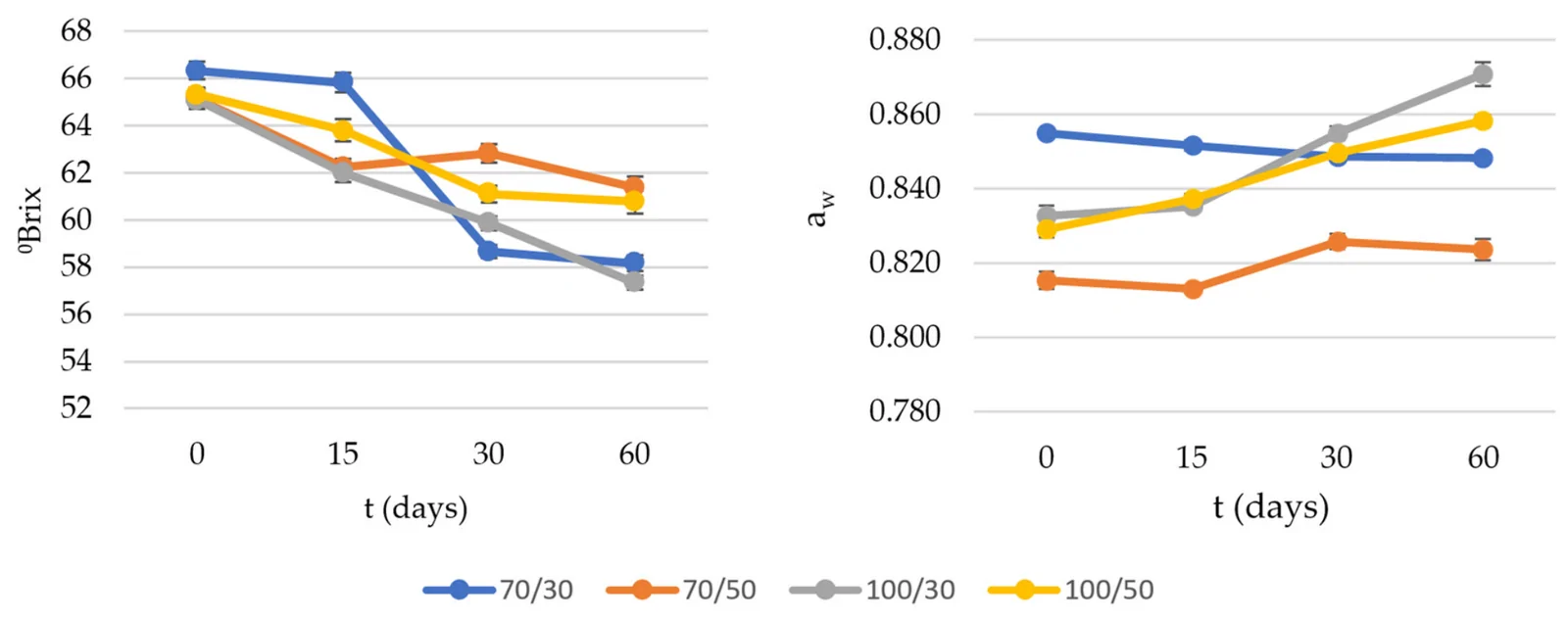
Evolution of total soluble solids (°Brix) and water activity (a_w_) of the four persimmon pastes during refrigerated storage; 70/30: processed at 70 °C with 30% sugar; 70/50: processed at 70 °C with 50% sugar; 100/30: processed at 100 °C with 30% sugar; 100/50: processed at 100 °C with 50% sugar.

**Figure 2:**
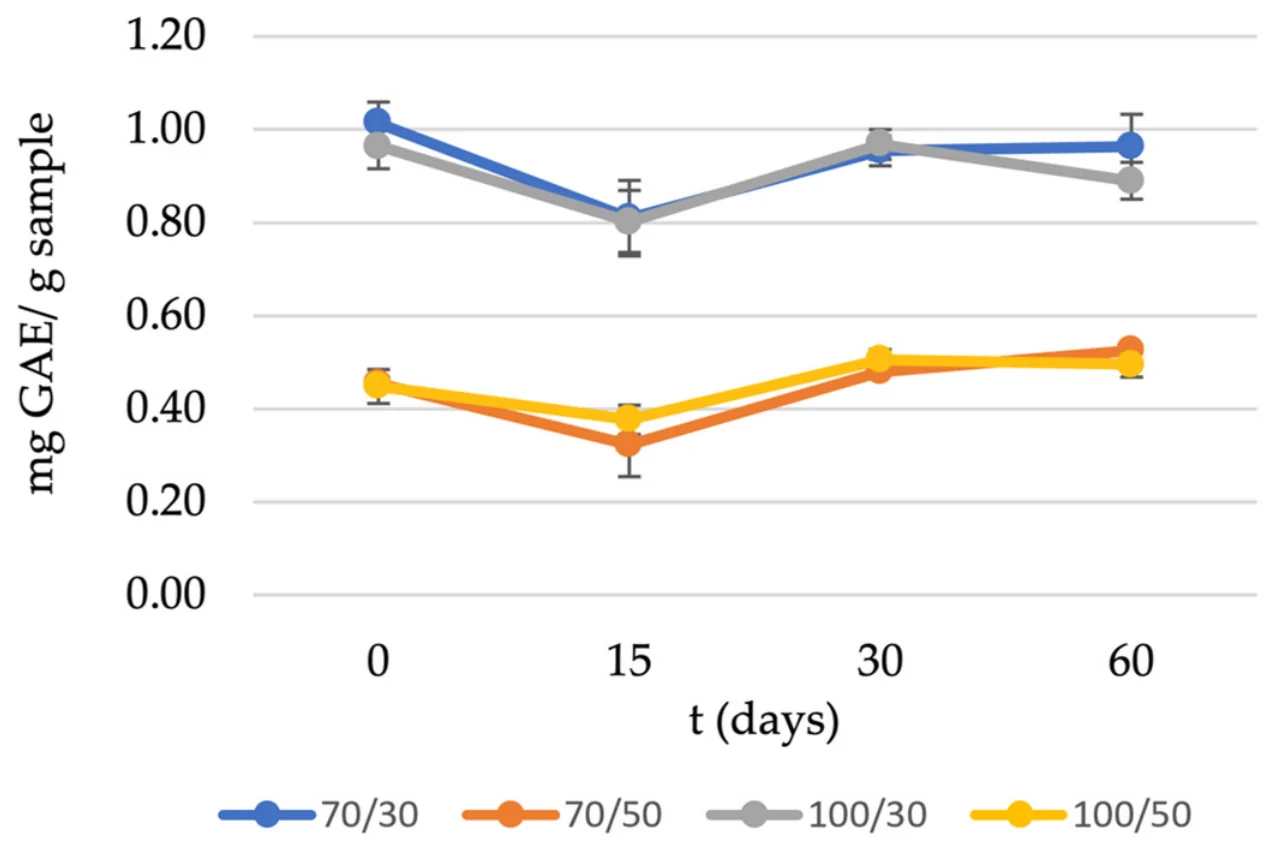
Evolution of soluble tannin content (mg gallic acid equivalent (GAE)/g sample) in the four persimmon pastes during refrigerated storage; 70/30: processed at 70 °C with 30% sugar; 70/50: processed at 70 °C with 50% sugar; 100/30: processed at 100 °C with 30% sugar; 100/50: processed at 100 °C with 50% sugar.

**Figure 3:**
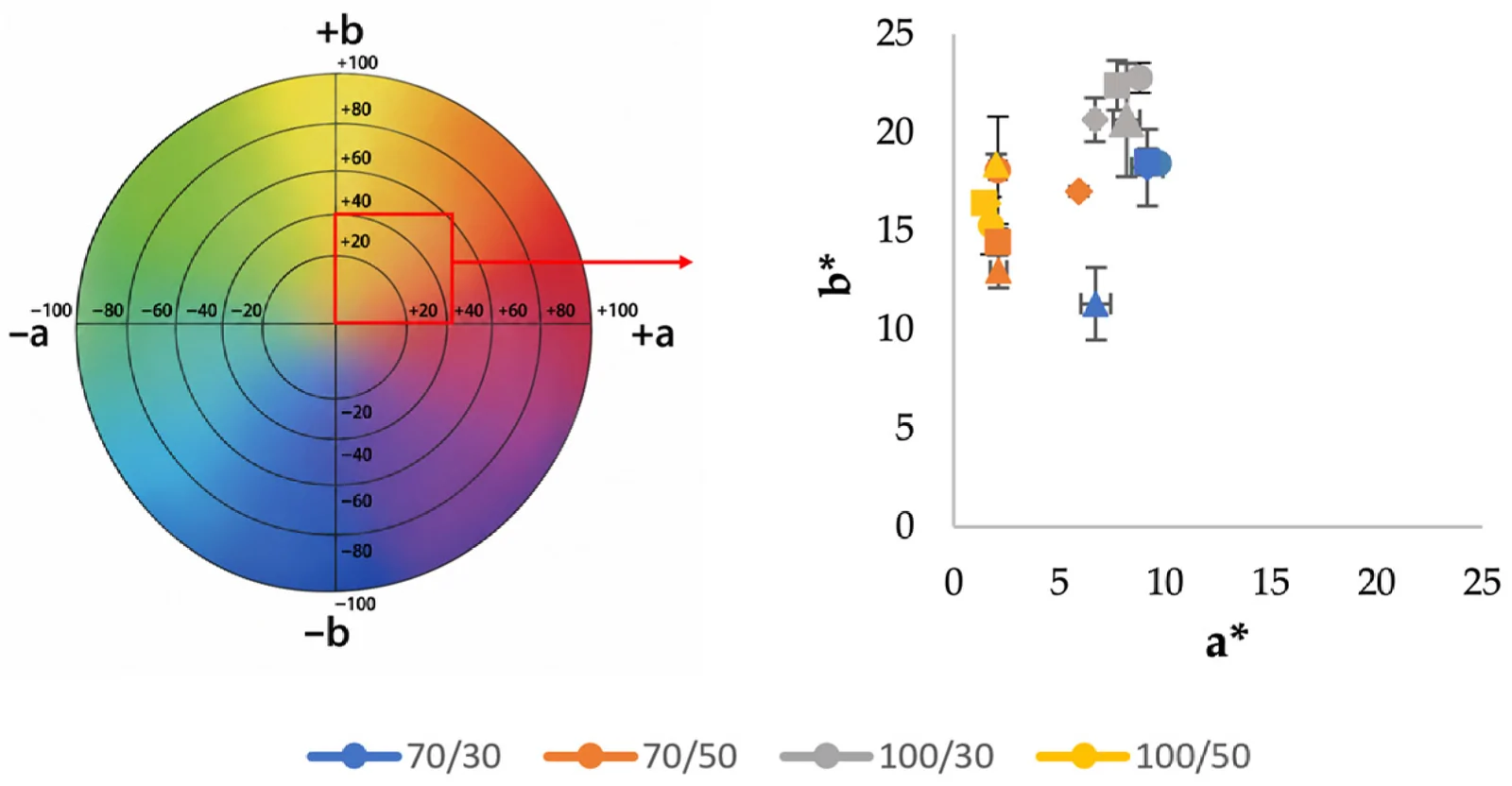
Position in the chromatic diagram of the four persimmon pastes during refrigerated storage; 70/30: processed at 70 °C with 30% sugar; 70/50: processed at 70 °C with 50% sugar; 100/30: processed at 100 °C with 30% sugar; 100/50: processed at 100 °C with 50% sugar. ●: storage time 0 days; ▲: 15 days; ■: 30 days; ♦: 60 days.

**Figure 4:**
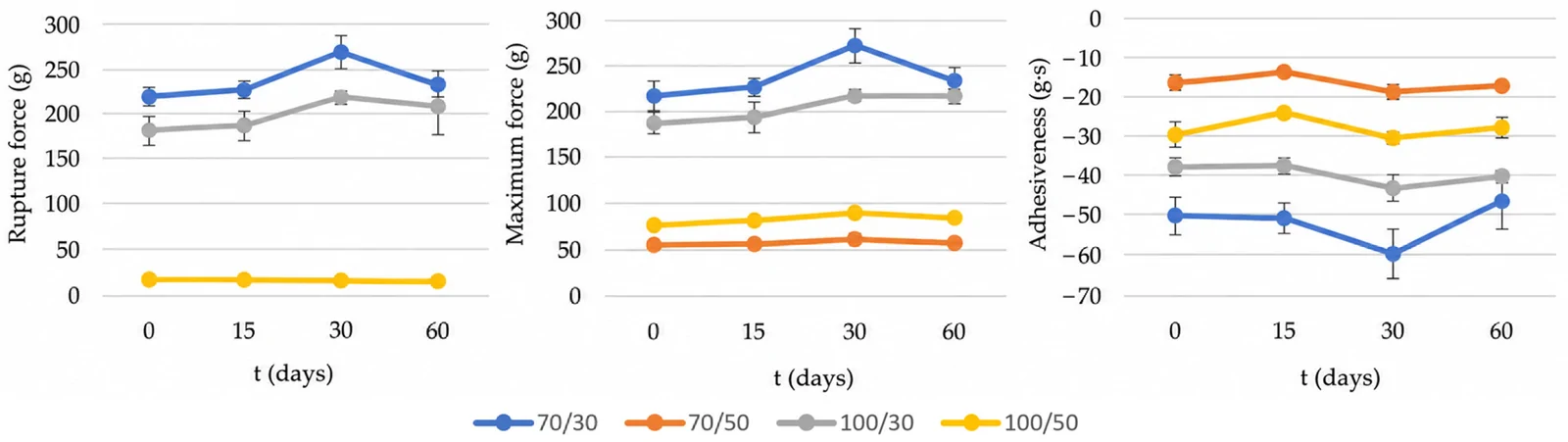
Evolution of texture parameters (rupture force, maximum force and adhesiveness) in the four persimmon pastes during refrigerated storage; 70/30: processed at 70 °C with 30% sugar; 70/50: processed at 70 °C with 50% sugar; 100/30: processed at 100 °C with 30% sugar; 100/50: processed at 100 °C with 50% sugar.

**Figure 5:**
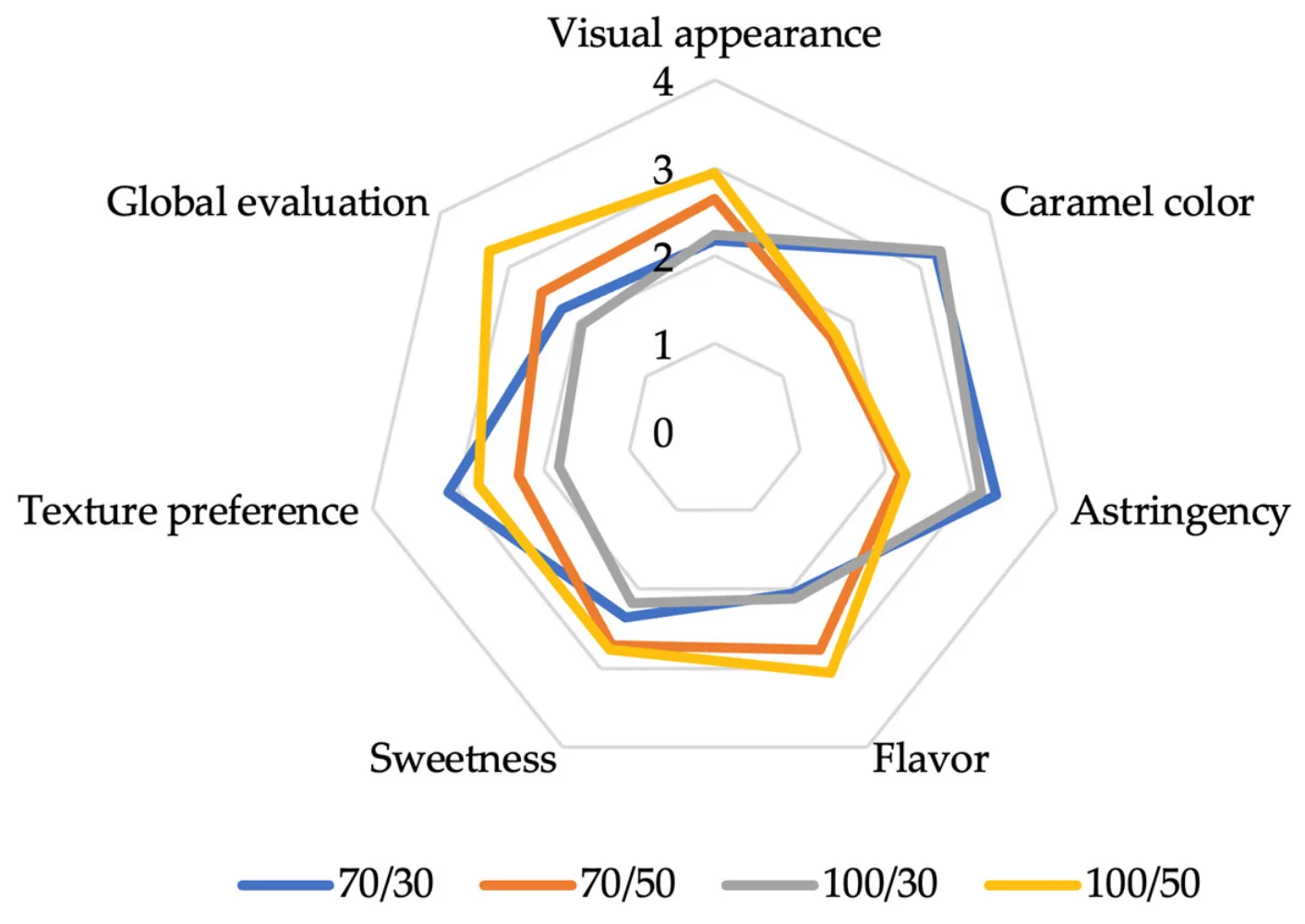
Ranking test; 70/30: processed at 70 °C with 30% sugar; 70/50: processed at 70 °C with 50% sugar; 100/30: processed at 100 °C with 30% sugar; 100/50: processed at 100 °C with 50% sugar.

**Figure 6:**
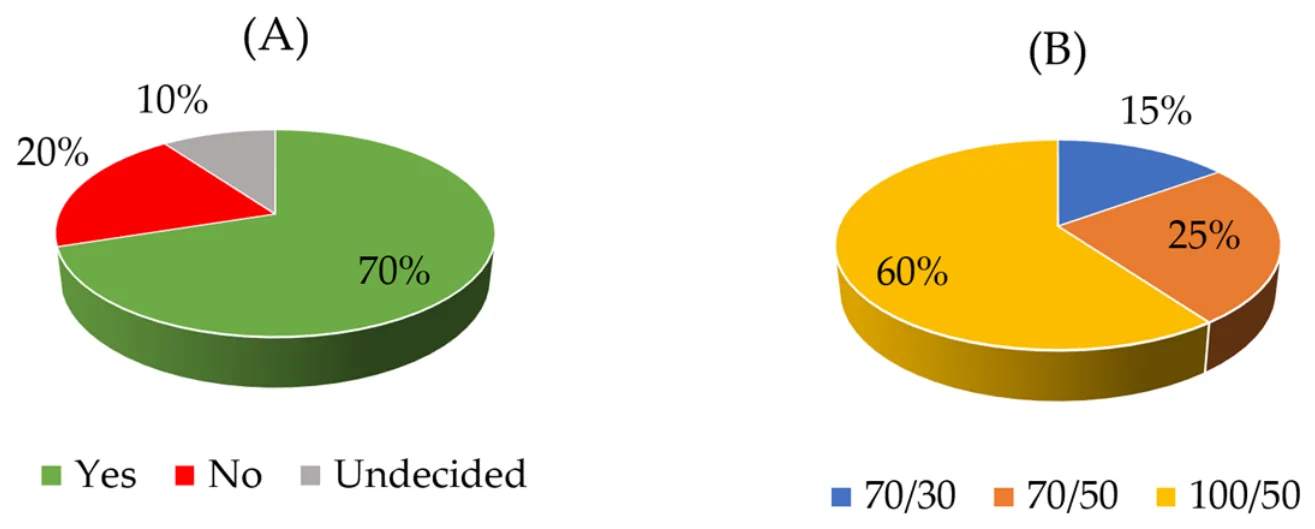
Purchase intention (**A**) of the panelists and product selected for purchase (**B**); 70/30: processed at 70 °C with 30% sugar; 70/50: processed at 70 °C with 50% sugar; 100/50: processed at 100 °C with 50% sugar.

**Table 1:** pH values of the four persimmon pastes during refrigerated storage; 70/30: processed at 70 °C with 30% sugar; 70/50: processed at 70 °C with 50% sugar; 100/30: processed at 100 °C with 30% sugar; 100/50: processed at 100 °C with 50% sugar.

Storage Time (Days)	70/30	70/50	100/30	100/50
0	3.96 ± 0.02 ^dA^	3.78 ± 0.01 ^cB^	3.87 ± 0.06 ^dAB^	3.69 ± 0.01 ^dC^
15	4.12 ± 0.04 ^bB^	3.96 ± 0.01 ^bC^	4.28 ± 0.03 ^bA^	3.97 ± 0.02 ^cC^
30	4.24 ± 0.01 ^aC^	4.15 ± 0.01 ^aD^	4.34 ± 0.02 ^aB^	4.43 ± 0.02 ^aA^
60	4.03 ± 0.01 ^cC^	3.97 ± 0.03 ^bD^	4.15 ± 0.01 ^cB^	4.18 ± 0.01 ^bA^

Different lowercase letters within each column indicate significant differences (*p* < 0.05) for the same sample as a function of storage time. Different uppercase letters within each row indicate significant differences (*p* < 0.05) among samples at the same storage time.

**Table 2:** Evolution of color attributes (lightness, hue angle, and chroma) of the four persimmon pastes during refrigerated storage. Color differences (ΔE*) are expressed relative to the initial storage time; 70/30: processed at 70 °C with 30% sugar; 70/50: processed at 70 °C with 50% sugar; 100/30: processed at 100 °C with 30% sugar; 100/50: processed at 100 °C with 50% sugar.

Sample	t (Days)	L*	h*	C*	ΔE*
70/30	0	36.0 ± 0.4 ^dC^	62.2 ± 0.5 ^bC^	20.8 ± 0.1 ^aB^	-
	15	41.5 ± 1.7 ^aB^	59.1 ± 1.6 ^cD^	13.2 ± 1.9 ^bC^	9.5
	30	38.1 ± 0.3 ^cD^	63.6 ± 0.6 ^aD^	20.7 ± 0.6 ^aB^	2.2
	60	39.2 ± 1.6 ^bB^	63.3 ± 0.7 ^aD^	20.4 ± 2.1 ^aA^	3.3
70/50	0	42.3 ± 2.3 ^aA^	83.4 ± 0.5 ^aA^	18.2 ± 2.7 ^aC^	-
	15	42.8 ± 2.3 ^aB^	80.7 ± 1.3 ^bB^	13.2 ± 0.9 ^bC^	5.1
	30	42.6 ± 0.7 ^aB^	81.8 ± 0.2 ^bB^	14.6 ± 0.7 ^cD^	3.6
	60	36.6 ± 0.2 ^bC^	70.8 ± 0.2 ^cC^	18.0 ± 0.2 ^aB^	7.0
100/30	0	42.2 ± 0.5 ^cA^	68.8 ± 0.3 ^bB^	24.4 ± 0.8 ^aA^	-
	15	46.0 ± 1.6 ^bA^	68.3 ± 1.3 ^bC^	22.2 ± 2.9 ^abcA^	4.7
	30	45.6 ± 0.6 ^bA^	70.9 ± 0.7 ^bC^	23.7 ± 1.2 ^abA^	3.6
	60	47.9 ± 0.8 ^aA^	72.0 ± 0.4 ^aB^	21.7 ± 1.1 ^cA^	6.4
100/50	0	38.7 ± 1.6 ^bB^	83.4 ± 0.8 ^cA^	15.4 ± 1.4 ^bD^	-
	15	36.5 ± 0.2 ^cC^	83.7 ± 0.7 ^bcA^	18.5 ± 0.5 ^aB^	3.7
	30	38.9 ± 0.1 ^bC^	85.0 ± 0.6 ^aA^	16.5 ± 0.1 ^bC^	1.2
	60	40.6 ± 0.6 ^aB^	84.4 ± 0.5 ^abA^	16.5 ± 0.5 ^bC^	2.2

Different lowercase letters within each column indicate significant differences (*p* < 0.05) for the same sample as a function of storage time. Different uppercase letters within each row indicate significant differences (*p* < 0.05) among samples at the same storage time.

## Data Availability

The original contributions presented in the study are included in the article, further inquiries can be directed to the corresponding author.
